# Seeking to humanize intensive care

**DOI:** 10.5935/0103-507X.20170003

**Published:** 2017

**Authors:** Gabriel Heras La Calle, Mari Cruz Martin, Nicolas Nin

**Affiliations:** 1 Hospital Universitario de Torrejón - Torrejón de Ardoz, Madrid, Spain.; 2 Proyecto HU-CI Humanizando los Cuidados Intensivos - Spain.; 3 Hospital Español - Montevideo, Uruguay.

## Introduction

The scientific and technical evolution of critical patient care has dramatically improved clinical practice and survival, but this progress has not been matched equally in the more human aspects of critical patient care. In many cases, the organizational and architectural characteristics of intensive care units (ICU) make them hostile environments for patients and their families and even for the professionals themselves.^(1)^

In a humanized organization, there is a personal and collective commitment to humanizing the relevant reality, relationships, behaviors, environment, and individuals, especially when the organization is aware of the vulnerability of others and the patient's need for help.

Many strategic lines can be considered in the context of humanizing ICUs, and all of these approaches allow a wide margin for improvement. Seeking excellence requires a change of attitude and a commitment to positioning the person as the central axis of health care.

**Figure 1:**
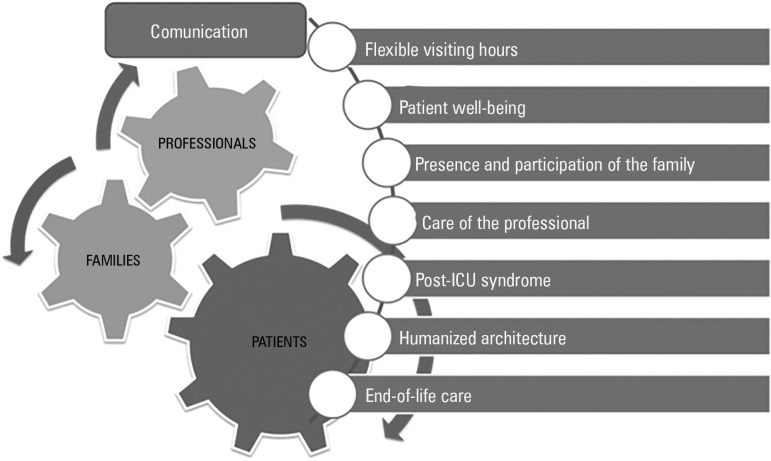
Conceptual framework for the humanization of critical care. ICU - intensive care unit.

Within the *Proyecto HU-CI: Humanizando los Cuidados Intensivos* [HU-IC Project: Humanizing Intensive Care], a conceptual framework has been designed with the objective of developing specific actions that contemplate humanization as a transverse dimension of quality. These areas of work cover aspects related to visitation schedules, communication, patient well-being, family participation in care, professional exhaustion syndrome, post-ICU syndrome, humanized architecture and infrastructure, and care at the end of life (Figure 1). All of these areas of focus hold offering excellent intensive care as a common objective, not only from a technical point of view but also from a human point of view, with the professional as the engine of change.

## Flexibility of visiting hours

Historically, policies concerning family visits to patients admitted to the ICU have followed a restrictive model based on the view that this approach favors care and facilitates the work of the professionals. However, the real foundation of this policy is tradition and a lack of critical reflection on its drawbacks.^(2)^ Families demand more time and the possibility to coordinate visits with their personal and work obligations.^(3)^ The experience of some units, such as pediatric and neonatal ICUs, in which the family is considered to be fundamental for comprehensive patient care, has led to the need to consider other models.^(4)^ At present, flexible visitation schedules and the establishment of "open doors" in the ICU are both possible and beneficial for patients, relatives, and professionals.^(5)^ Extending the model requires learning from the positive experiences of some units, as well as professional participation, training, and changes in attitudes and habits that allow an open modification of the visitation policy, which can be adapted to the idiosyncrasies of each unit. The figure of the "primary caregiver" can favor the presence of relatives who are adapted to the individual needs of each patient and his or her environment.

## Communication

In the ICU, teamwork, which is essential in any type of health care, requires effective communication, among other elements.^(6)^ Transfers of information (shift changes, duty changes, transfers of patients to other units or services, etc.), during which not only information but also responsibility is exchanged, are frequent and require structured procedures that make them more effective and secure. Regarding this important process, adequate leadership and the use of tools that facilitate multidisciplinary participation are key elements in improving communication.^(7)^

Conflicts among the professionals who make up ICU teams are frequent and are caused in many cases by communication failures. These conflicts threaten the team concept, directly influence the well-being of the patient and the family, generate wear and tear, and negatively impact results.^(8)^ Training in both non-technical skills and support strategies can promote team cohesion.

Information is one of the main needs expressed by patients and relatives in ICUs.^(9)^ When treating the critical patient, who is often incapacitated, the right to information is frequently transferred to his/her relatives. Informing adequately in situations of great emotional burden requires communication skills, for which many professionals have not received specific training. Effective communication with patients and families fosters a climate of trust and respect and facilitates joint decision-making. In general, no specific policies outline how to carry out the informative process in the ICU, with information often provided once each day, without adapting to the specific needs of patients and their relatives. In addition, joint physician-nurse information is still rarely available.

The inability of many critical patients to communicate or speak generates negative feelings, which are an important source of stress and frustration for patients, families, and professionals.^(10)^ The use of augmentative and alternative communication systems should be incorporated systematically as a tool to improve communication for these patients.^(11)^

## Patient well-being

Many factors cause suffering and discomfort for critical patients. Patients suffer from pain, thirst, cold and heat, and difficulty resting due to excessive noise or illumination; they also have limited communication or mobility, often because of the use of unnecessary constraints.^(12)^ The assessment and control of pain, dynamic sedation appropriate to the patient's condition, and the prevention and management of acute delirium are indispensable parts of improving the comfort of patients.^(13)^ In addition to physical causes of suffering, psychological and emotional suffering can be very important. Patients experience feelings of loneliness, isolation, fear, dependency, uncertainty due to lack of information, incomprehension, and loss of identity, intimacy, and dignity, among others.^(14)^ The evaluation and support of these needs should be considered as a key element of providing quality care.^(15)^ Ensuring adequate training of professionals and promoting measures that aim to treat or mitigate these symptoms and ensure the well-being of patients is a main objective in the care of the critical patient.

## Family presence and participation in the care

Family members present a high prevalence of post-traumatic stress, anxiety, and depression. Although family members generally wish to participate in the patient's care and many would consider staying with their loved ones, especially at times of high vulnerability, the presence and participation of family members in the ICU is very limited. The barriers to such participation have focused on the possible psychological trauma and anxiety that can be generated for the family, interference with procedures, distraction, and the possible impact on the medical team.

If clinical conditions allow it, families who desire to participate could collaborate on some aspects of basic care (grooming, meal management, or rehabilitation) under the training and supervision of health professionals. Giving family members the opportunity to contribute to the recovery of the patient can have positive effects on the patient, the family members, and the professional, by reducing emotional stress and facilitating closeness and communication among the involved parties.

Although the available studies are inconclusive, the presence of relatives during certain procedures has not been associated with negative consequences and is accompanied by changes in the attitude of the professionals, such as greater concern among professionals in relation to privacy, dignity, and pain management during the witnessed procedures. The presence of relatives is also associated with greater satisfaction among family members and increased acceptance of the situation, which favors the process of mourning.^(16)^

## Care of the professional

"Burnout syndrome" or "professional exhaustion syndrome" is a professional disease that is characterized by 3 classic symptoms: emotional exhaustion, depersonalization, and feelings of low professional self-esteem.^(17,18)^ This syndrome impacts professionals at the personal and professional levels, resulting in post-traumatic stress syndrome, serious psychological disorders, and even suicide. Burnout syndrome also influences the quality of care, patient outcomes, and patient satisfaction and is related to the turnover of professionals in organizations.

Contributing factors include individual personal characteristics, as well as environmental and organizational factors. These factors, directly or through intermediate syndromes, such as "moral distress," which is the perception of offering inappropriate care, or "compassion fatigue," can lead to professional exhaustion syndrome.^(19)^

Recently, scientific societies have sought to improve the visibility of this syndrome, offering recommendations to reduce its appearance and mitigate its consequences and establishing specific strategies that allow a suitable response to the physical, emotional, and psychological needs of intensivist professionals, which are derived from their dedication and effort in performing their work.^(20)^

## Detection, prevention, and management of post-intensive care unit syndrome

Post-intensive care syndrome, which was described recently, affects a significant number of patients (30 to 50%) after a critical illness. This syndrome is characterized by physical symptoms (such as persistent pain, weakness acquired in the ICU, malnutrition, pressure ulcers, sleep disturbances, and the need to use devices), neuropsychological symptoms (cognitive deficits, such as disorders of memory, attention, and mental processing speed), or emotional symptoms (anxiety, depression, and post-traumatic stress) and can also affect the patient's family members, causing social problems.^(21)^ The medium- and long-term consequences of post-intensive care syndrome impact the quality of life of patients and their families. Multidisciplinary teams that include specialists in rehabilitation and physiotherapy, nurses, psychologists, psychiatrists, occupational therapists, and speech therapists facilitate the continuous care that is necessary to support these needs.

## Humanized architecture and infrastructure

The physical environment of the ICU should allow the healthcare process to proceed in a healthy environment, which helps to improve the physical and psychological states of patients, professionals, and family members. Published guidelines (Evidence Based Design) seek to reduce stress and promote comfort by focusing on the architectural and structural improvements of ICUs that are appropriate to both users and workflows. These guidelines contemplate environmental conditions, including light, temperature, acoustics, materials and finishes, furniture, and decor. These modifications can positively influence feelings and emotions, favoring human spaces that are adapted to the functionality of the units. Other spaces, such as waiting rooms, should be redesigned so that they become "living rooms" and offer greater comfort and functionality to the patients' families.

## End-of-life care

Palliative and intensive care are not mutually exclusive options but should coexist throughout the process of critical patient care.^(22)^ Although the fundamental objective of intensive care is to restore the situation prior to the patient's admission, this outcome is sometimes not possible and must be modified dynamically, with the aim of reducing suffering and offering the best care, especially at the end of life. Palliative care seeks to provide comprehensive care for the patient and his/her environment, with the intention of allowing a death free of discomfort and suffering for the patient and his or her family members, in accordance with their clinical, cultural, and ethical wishes and standards. The decision to limit vital support, which is made frequently in the critically ill patient, should be made following the guidelines and recommendations established by scientific societies.^(23,24)^ Such limits should be integrated into a comprehensive palliative care plan, in a multidisciplinary manner, with the objective of meeting the needs of the patients and his or her family members, including physical, psychosocial, and spiritual needs.^(25)^ The existence of specific protocols and the periodic evaluation of the care offered constitute basic requirements. The complex decisions that are made when caring for critical patients at the end of life can lead to discrepancies among health professionals and discrepancies between health professionals and patient families. The professionals must have the necessary skills and tools to solve these conflicts by incorporating open and constructive discussion into these situations as coping strategies to reduce the emotional burden derived from them.

## Conclusions

To humanize is to seek excellence from a multidimensional point of view, addressing all facets of a person rather than clinical needs alone. This approach increases closeness and tenderness, with self-criticism and the capacity for improvement. Intensive care units and critical care professionals have a moral commitment to lead the change.
